# The Impact of Endothelial Progenitor Cells on Restenosis after Percutaneous Angioplasty of Hemodialysis Vascular Access

**DOI:** 10.1371/journal.pone.0101058

**Published:** 2014-06-25

**Authors:** Chih-Cheng Wu, Po-Hsun Huang, Chao-Lun Lai, Hsin-Bang Leu, Jaw-Wen Chen, Shing-Jong Lin

**Affiliations:** 1 Cardiovascular center, National Taiwan University Hospital, Hsinchu Branch, Hsinchu, Taiwan; 2 Division of Cardiology, Taipei Veterans General Hospital, Taipei, Taiwan; 3 Department of Medical Research and Education, Taipei Veterans General Hospital, Taipei, Taiwan; 4 Healthcare and Management Center, Taipei Veterans General Hospital, Taipei, Taiwan; 5 School of Medicine, National Taiwan University, Taipei, Taiwan; 6 Department of Medicine, National Yang-Ming University, Taipei, Taiwan; 7 Institute of Clinical Medicine, National Yang-Ming University, Taipei, Taiwan; 8 School of Medicine, National Yang-Ming University, Taipei, Taiwan; 9 Cardiovascular Research Center, National Yang-Ming University, Taipei, Taiwan; 10 Institute and Department of Pharmacology, National Yang-Ming University, Taipei, Taiwan; University of Milan, Italy

## Abstract

**Objective:**

We prospectively investigate the relation between baseline circulating endothelial progenitor cells and the subsequent development of restenosis after angioplasty of hemodialysis vascular access.

**Background:**

Effect of angioplasty for hemodialysis vascular access is greatly attenuated by early and frequent restenosis. Circulating endothelial progenitor cells (EPCs) play a key role in vascular repair but are deficient in hemodialysis patients.

**Method:**

After excluding 14 patients due to arterial stenosis, central vein stenosis, and failed angioplasty, 130 patients undergoing angioplasty for dysfunctional vascular access were prospectively enrolled. Flow cytometry with quantification of EPC markers (defined as CD34^+^, CD34^+^KDR^+^, CD34^+^KDR^+^CD133^+^) in peripheral blood immediately before angioplasty procedures was used to assess circulating EPC numbers. Patients were followed clinically for up to one year after angioplasty.

**Results:**

During the one-year follow-up, 95 patients (73%) received interventions for recurrent access dysfunction. Patients in the lower tertile of CD34^+^KDR^+^ cell count had the highest restenosis rates (46%) at three month (early restenosis), compared with patients in the medium and upper tertiles of CD34^+^KDR^+^ cell count (27% and 12% respectively, p = 0.002). Patients in the lower tertile of CD34^+^KDR^+^ cell count received more re-interventions during one year. Patients with early restenosis had impaired EPC adhesive function and increased senescence and apoptosis. In multivariate analysis, the CD34^+^KDR^+^ and CD34^+^KDR^+^CD133^+^ cell counts were independent predictors of target-lesion early restenosis.

**Conclusion:**

Our results suggest that the deficiency of circulating EPCs is associated with early and frequent restenosis after angioplasty of hemodialysis vascular access.

## Introduction

A functioning vascular access greatly influences the survival and quality of life of patients undergoing hemodialysis. Vascular accesses are subject to failure and the underlying pathology is usually a stenosis due to venous intimal hyperplasia. [1] Endovascular interventions are useful in restoring the function of vascular access. [2] Nonetheless, their benefits are attenuated by a high restenosis rate, far more aggressive than that of arterial lesions in non-uremic patients. Physiological and anatomical differences between arteries and veins, continuous hemodynamic stress, repeated puncture, uremia and endothelial dysfunction have all been proposed as possible causes. [1] However, in all patients, it is not precisely known how much the listed factors contribute to the high restenosis rates.

Maintenance of the integrity and function of the endothelium has been shown to play a pivotal role in the prevention of restenosis after angioplasty. [3] Accumulating evidence suggests that circulating endothelial progenitor cells (EPCs) incorporate into sites of endothelial denudation. [4] The circulating EPCs reflect not only repair capacity but also the health of the endothelium. [5] Clinical studies have shown that circulating EPC numbers are decreased and associated with vascular events in hemodialysis patients. [6], [7] However, limited data are available about the role of EPCs for venous intimal hyperplasia in hemodialysis patients.

Accordingly, we conducted this prospective study to evaluate the impact of circulating EPC number and function on the outcome of vascular access.

## Methods

### Ethical statements

The study was based on the Declaration of Helsinki (edition 6, revised 2000). Written informed consent was obtained from all study participants, and the study was approved by the Institutional Review Board of our hospital (Hsinchu General Hospital Institutional Review Board, No. HCGH99G005 and National Taiwan University Hospital Hsinchu Branch Institutional Review Board, No. HCGH100G003).

### Study participants

From January 2010 to July 2011, a series of patients with dysfunctional hemodialysis vascular access scheduled for percutaneous intervention were prospectively enrolled. Patients were referred based on one or more of the following criteria: clinical signs suggesting fistula dysfunction, reduction of flow rate and increased venous pressure during dialysis. Participants had to have received regular dialysis treatment for at least 6 months without clinical evidence of acute or chronic inflammation, recent myocardial infarction, unstable angina, or circulatory congestion. According to the same criteria, 26 patients who had normal functioning vascular access for at least two years were invited as the uremic control group. Another 30 non-uremic patients who received cardiac catheterization examination with patent coronary arteries were invited as the non-uremic control group.

### Study protocol

Patients eligible for this study were scheduled for diagnostic fistulography and angioplasty on a mid-week non-dialysis day. Baseline characteristics and blood samples were collected on the morning of fistulography. After diagnostic fistulography or angioplasty, patients with insignificant stenosis (less than 50% diameter stenosis), thrombosed fistulas, arterial side stenosis, central vein lesions, or those who failed to obtain anatomic success after angioplasty were excluded. Diagnostic fistulography and angiograms of the angioplasty procedures were independently reviewed by another expert angiographer who was unaware of the patients’ clinical and analytic data. The degree of stenosis was evaluated by two orthogonal planes and the greatest degree of stenosis was used for subsequent anatomical measurements. Anatomic measurements were made with use of a calibrated reference maker or computer-assisted edge detection software within the angiographic imaging system. The reference vessel was defined as an adjacent segment of normal vein located upstream from the target lesion. The degree of stenosis was reported as the maximum diameter reduction compared to the reference vessel diameter.

### Laboratory methods

Blood samples were drawn after a 12 hour overnight fast and cessation of medications before diagnostic procedures. Plasma biochemical parameters were analyzed by standard laboratory methods. Assessment of the circulating EPCs by flow cytometry was performed by researchers blinded to clinical data. [8]–[10] A volume of 1000-µL peripheral blood was incubated for 30 minutes in the dark with Allophycocyanin (APC)-conjugated monoclonal antibody against human KDR (R&D, Minneapolis, MN, USA), Phycoerythrin (PE)-conjugated monoclonal antibody against human CD133 (Miltenyi Biotec, Germany), and Fluorescein isothiocyanate (FITC)-conjugated monoclonal antibodies against human CD34 (Becton Dickinson Pharmingen, USA). After incubation, cells were lysed, washed with phosphate-buffered saline (PBS), and fixed in 2% paraformaldehyde before analysis. Each analysis included 150,000 events. The number of cells was normalized and expressed as a percentage (%) of cells and cells per 1×10^5^ mononuclear cells (MNC). To assess the reproducibility of EPC measurements, circulating EPCs were measured from 2 separate blood samples in 10 subjects, and there was a strong correlation between the two measurements (r = 0.90, P<0.001).

### Human EPC culture and functional studies

Peripheral blood samples for EPC culture were obtained from twenty of the study participants, ten from the early restenosis group (restenosis developed within three months after angioplasty) and ten from the late restenosis group (restenosis within 4–12 months after angioplasty), matched by age and types of vascular access. These samples were collected retrospectively while vascular accesses were functioning well after angioplasty. Total MNCs were isolated by density gradient centrifugation with Histopaque-1077 (Sigma, St. Louis, MO, USA). [11] Briefly, MNCs (5×10^6^) were plated in 2 ml endothelial growth medium (EGM-2 MV Cambrex, East Rutherford, NJ, USA) on fibronectin-coated 6-well plates. After 4 days of culturing, the medium was changed and non-adherent cells were removed; attached EPCs appeared elongated with a spindle shape. EPCs were collected and used for the functional assays in this study.

Fibronectin adhesion test were assessed as previous described. [12] Cellular aging was determined with a Senescence Cell Staining kit (Sigma). TUNEL assay (Terminal deoxynucleotidyl transferase mediated deoxyuridine triphosphate nick-end labeling) was performed using the In Situ Cell Death Detection kit (Roche Diagnostics, Basel, Switzerland) according to the instructions of the manufacturers (**Methods S1**).

### Follow-up and definitions

After the angioplasty procedure, all the participants of this study were prospectively followed for one year under the same protocol at respective hemodialysis centers. Medications of the participants were continued or adjusted according to their original indications but not for the maintenance of their vascular accesses. Follow-up surveillance included physical examination and dynamic venous pressure monitoring at each hemodialysis session, and transonic examination of access blood flow rate immediately after the intervention followed by monthly examinations. The referring nephrologists were blinded to the EPC levels of their patients. When abnormal clinical or hemodynamic parameters fulfilling the original referral criteria were detected, patients were referred for repeat fistulography and angioplasty as appropriate.

Anatomic success was defined as less than 30% residual stenosis. Clinical success was defined as an improvement from baseline in clinical or hemodynamic parameters indicative of access dysfunction. Success of the procedure was defined as the combination of anatomic and clinical success. Target-lesion restenosis was defined as more than 50% diameter reduction of the original target lesion. Primary patency of vascular access was defined as time until the next intervention on the access of any kind; secondary patency of vascular access was defined as time from the intervention until surgical revision or abandonment of the access.

### Statistical analysis

All data are presented as means ± standard deviations or percentages. Categorical data were compared using the Chi-square test with Yates’ correction and Fisher’s exact test as appropriate. Continuous variables were tested for a normal distribution by the Kolmogorov-Smirnov test. For normally distributed data, means between categories were compared by one-way analysis-of-variance. For non-normally distributed data, the Kruskal-Wallis test was used for comparison between categories. We made the assumption of three times relative risk of restenosis according to a previous study about the role of CD34^+^ cell counts on cardiovascular events in hemodialysis patients. [7] There were 39 restenosis events in the 130 patients enrolled in our study. With the assumption of a hazard ratio for restenosis = 3.0, alpha level = 0.05, and power = 80%, each group needs 16 restenosis events by the log rank test. Thus, we stratified our patients into tertiles to compare the relative risk or restenosis between tertiles of CD34^+^KDR^+^ cell counts. The primary patency of the whole access in each group was estimated by the Kaplan-Meier method and differences were assessed using the log-rank test. Proportions of patients with early restenosis, late restenosis, and no restenosis were compared by Chi-square test. Cox regression analysis was used for estimating the relative hazard of vascular access events by tertile of CD34^+^KDR^+^ cell count with subjects in the lower tertile of CD34^+^KDR^+^ cell count as the reference group. All variables with P<0.2 in the univariate analysis (including use of calcium channel blocker, side of access, location of access, nature of access, diameter of access and post-dilatation stenosis) and traditional cardiovascular risk factors (including age, hypertension, diabetes mellitus, dyslipidemia, and smoking) were entered into a multivariate analysis to determine independent predictors. A P value of less than 0.05 was considered to be statistically significant. Statistical analysis was performed with the use of SPSS software, version 20.0 for Windows.

## Results

### Baseline characteristics of study participants

One hundred and forty-four patients with dysfunctional vascular access were enrolled prior to the diagnostic procedures. After diagnostic fistulography and angioplasty, fourteen patients were excluded: five due to central vein lesions; two due to arterial lesions, five due to thrombosed lesions, and two due to a failed angioplasty procedure. Therefore, the study group consisted of 130 patients. All patients underwent one-year clinical follow-up after the angioplasty procedure apart from six patients who died before the end of one-year.

**Figure 1:**
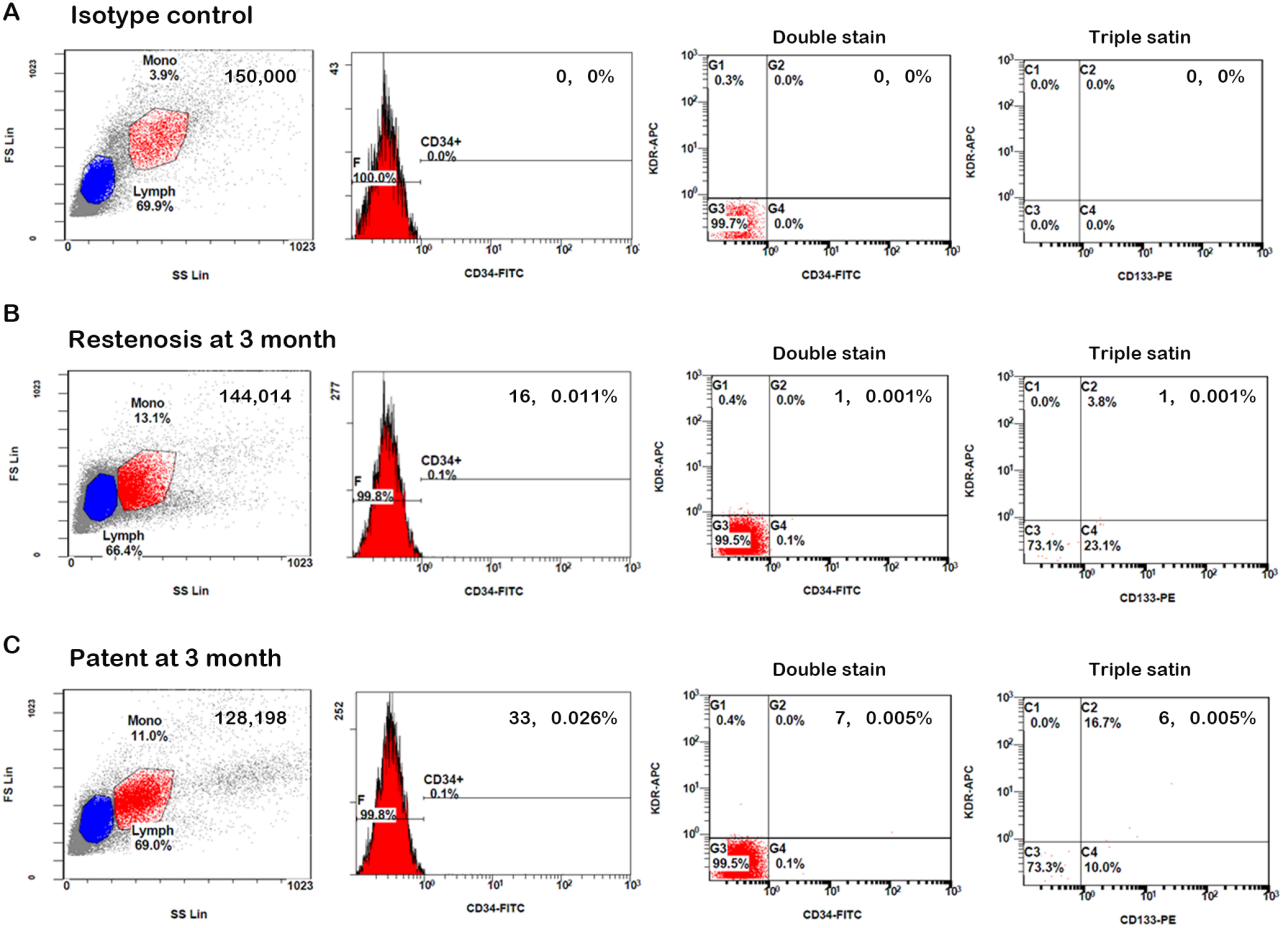
Representative flow cytometry analysis. Panels show mononuclear cells (MNCs) that were gated by forward/sideward scatter (FSC/SSC) in isotype controls (A), patients with restenosis (B), and patients without (C) restenosis at 3 months. The numbers of circulating endogenous progenitor cells (EPCs) were defined as CD34^+^, CD34^+^KDR^+^, and CD34^+^KDR^+^CD133^+^, respectively.

**Figure 2:**
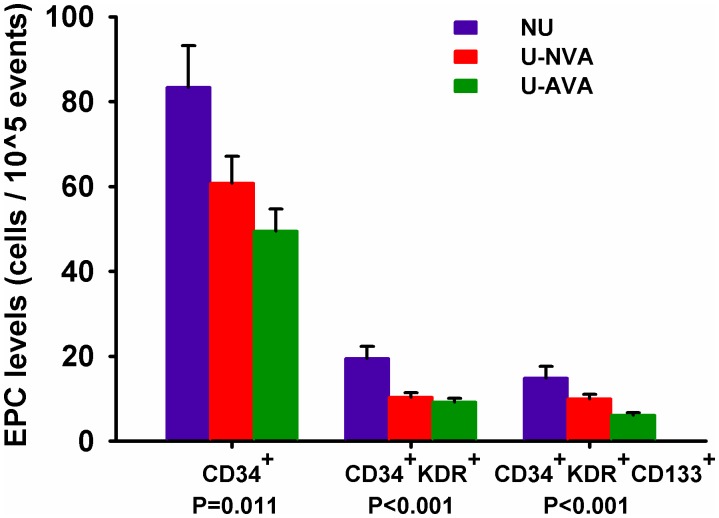
Circulating EPC counts between different groups. Comparisons of EPCs counts between the non-uremic controls (NU), uremic controls (normal vascular access function without interventions in previous two years, U-NVA), and uremic patients with abnormal vascular access function referred for interventions (U-AVA). (Values presented as mean ± standard error).

As shown in **Figure 1**, the circulating EPCs were gated with monocytes and defined as CD34^+^, CD34^+^KDR^+^, and CD34^+^KDR^+^CD133^+^, respectively. Compared to the non-uremic control group, patient with dysfunctional vascular access had significantly lower CD34^+^ cells (50±60 vs. 83±54 cells/10^5^ MNCs, p = 0.014), CD34^+^KDR^+^ cells (9±9 vs. 20±15 cells/10^5^ MNCs, p<0.001), and CD34^+^KDR^+^CD133^+^ cells (6±6 vs. 15±15, p<0.001). Compared to the uremic control group, patients with dysfunction vascular access had a trend of lower CD34^+^ cell counts (50±60 vs. 61±31 cells/10^5^ MNCs, p = 0.66), CD34^+^KDR^+^ cell counts (9±9 vs. 10±5 cells/10^5^ MNCs, p = 0.88), and CD34^+^KDR^+^CD133^+^ cell counts (6±6 vs. 10±5, p = 0.08) but the difference didn’t reach statistical significance (**Figure 2**).

### Endothelial progenitor cell counts and baseline variables

Participants with abnormal vascular access function were stratified into tertiles according to their baseline CD34^+^KDR^+^ cell count (lower, <4.0; medium, 4.0 to 9.0; upper, >9.0 cells/10^6^ MNCs). The baseline characteristics of study participants and vascular accesses were shown in **Table 1**. In terms of baseline characteristics, only albumin level and white cell count were associated with tertile of baseline CD34^+^KDR^+^ cell count. In addition to CD34^+^KDR^+^ cells, we also estimated surface marker CD133^+^ cells, which were expressed as a subfraction of immature EPCs. Increased age and white cell count were associated with a higher CD34^+^KDR^+^CD133^+^ cell count.

**Table 1:** Baseline characteristics of patients and vascular accesses.

		Tertiles of CD34^+^KDR^+^ Cell Count			
Characteristic	Total (N = 130)	Lower (N = 43)	Medium (N = 44)	Upper (N = 43)	P value
Age (yr)	66±13	71±12	65±12	63±14	0.06
Gender (men/women)	47/83	14/29	20/24	13/30	0.31
**Risk factors**					
Hypertension (%)	76(58%)	24(56%)	26(59%)	26(60%)	0.90
Diabetes (%)	48(37%)	15(35%)	17(39%)	16(37%)	0.94
Dyslipidemia (%)	23(18%)	11(26%)	4(9%)	8(19%)	0.13
Current smoker (%)	15(11%)	5(11%)	6(14%)	4(9%)	0.71
Cardiovascular disease (%)	26(20%)	12(28%)	8(18%)	6(14%)	0.25
**Biochemical data**					
Cholesterol (mg/dl)	166±40	170±38	164±48	163±33	0.71
Triglycerides (mg/dl)	161±108	177±106	147±99	159±117	0.51
Albumin (g/dl)	3.9±0.5	3.8±0.5	3.8±0.4	4.1±0.4	0.004
Hemoglobin (g/dl)	10.8±1.4	10.7±1.7	10.8±1.3	10.9±1.2	0.88
WBC (10^3^/µL)	6.7±2.0	7.2±2.7	5.8±1.4	7.1±1.5	0.01
Calcium (mg/dl)	9.4±1.2	9.1±1.7	9.6±0.9	9.6±0.9	0.16
Phosphate (mg/dl)	4.4±1.3	4.3±1.3	4.4±1.5	4.6±1.1	0.53
Kt/V	1.46±0.30	1.28±0.30	1.62±0.30	1.63±0.27	0.07
**Medications**					
Anti-platelet	50(43%)	15(38%)	17(47%)	18(46%)	0.64
Nitrates	22(19%)	9(23%)	7(19%)	6(15%)	0.72
β-blocker	23(50%)	7(18%)	7(19.4%)	9(23%)	0.82
Calcium blocker	34(30%)	11(28%)	12(33%)	11(28%)	0.83
ACEI/ARB	23(20%)	6(15%)	8(22.2%)	9(23%)	0.62
Lipid-lowering agents	18(16%)	5(13%)	5(14%)	8(21%)	0.58
Erythropoietin (U/kg/week)	80±36	78±40	79±37	82±34	0.94
**Access/lesion**					
Shunt age (month)	48±44	42±31	58±57	45±40	0.30
Prosthetic graft	70(54%)	20(47%)	22(50%)	28(65%)	0.18
Upper arm access	19(15%)	7(16%)	8(18%)	4(9%)	0.50
Right arm access	32(25%)	13(30%)	10(23%)	9(21%)	0.60
Diameter (mm)	7.2±1.3	7.1±1.2	7.3±1.4	7.2±1.2	0.76
Pre-stenosis (%)	73±15	71±15	75±15	72±14	0.47
Post-stenosis (%)	22±17	24±17	23±16	21±19	0.78
**CD34^+^KDR^+^ cells/10^5^MNCs**	9.2±9.4	2.3±1.2	6.3±1.3	19.1±10.4	<0.001

ACEI, angiotensin converting enzyme inhibitor; ARB, angiotensin receptor blocker; Kt/V, urea clearance; MNC, mononuclear cell; WBC, white blood cell.

P for ANOVA or Kruskal-Wallis test in continuous variables and p for Chi-square test in categorical variables.

### Incidence of vascular and clinical events (Table 2)

At the end of one year follow-up, 95 patients underwent a re-intervention; 94 of whom had a restenosis at the same location. All the patients with target lesion restenosis received re-interventions and were included in the re-intervention group as well. Nine patients lost their vascular access and six patients died during the follow-up period: three patients due to cardiovascular causes (myocardial infarction or sudden cardiac death) and the others due to non-cardiovascular causes.

**Figure 3:**
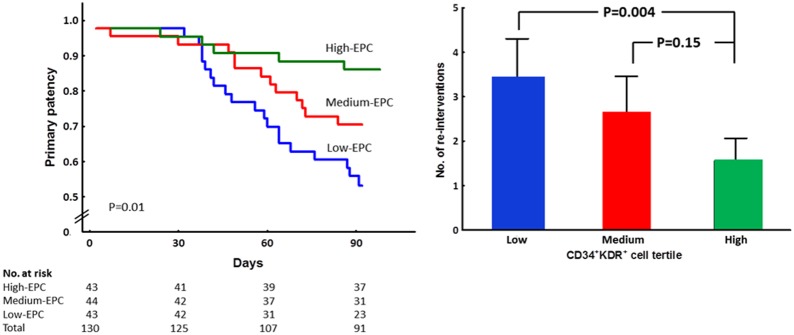
Kaplan-Meier analyses at three months and frequency of re-intervention at one year stratified by EPC tertiles. Left: The figure demonstrates the proportion of patients without target-lesion early restenosis according to their baseline circulating CD34^+^KDR^+^ cell count. Patients are divided into tertiles (low, medium, high) according to their baseline circulating CD34^+^KDR^+^ cell count. Right: Frequency of re-interventions at one year after angioplasty stratified by baseline circulating CD34^+^KDR^+^ cell count. (Values presented as mean ± standard error).

Because the majority of patients had target-lesion restenosis at the end of one-year follow-up, the vascular access events were further stratified according the presence and timing of target-lesion restenosis: early restenosis (within 3 months), late restenosis (between 4–12 months) and no restenosis (within 12 months). The same time frame was applied in the stratification of vascular access re-intervention. Patients in the lower tertile of CD34^+^KDR^+^ cell count had the highest rate of early restenosis and re-intervention. In contrast, patients in the highest tertile of CD34^+^KDR^+^ cell count possessed the highest proportions of late or no restenosis compared to the other two tertiles, although they did not achieve statistical significance. The discrepancy in cumulative primary patency rates between EPC groups was largest at three months and then declined as the follow-up period extended (**Table 2** **and** **Figure 3**).

**Table 2:** Vascular access and clinical events during follow-up period.

		Tertiles of CD34^+^KDR^+^ Cell Count			
Event	Total (N = 130)	Low (N = 43)	Medium (N = 44)	High (N = 43)	P value
**Vascular access event**					
Target-lesion restenosis					
Early restenosis	37(28%)	20(46%)	12(27%)	5(12%)	0.002
Late restenosis	57(44%)	14(33%)	19(43%)	24(56%)	0.09
No restenosis	36(28%)	9(21%)	13(30%)	14(33%)	0.46
Access re-intervention					
Early re-intervention	39(30%)	20(46%)	13(30%)	6(14%)	0.004
Late re-intervention	56(43%)	14(33%)	18(41%)	24(56%)	0.09
No re-intervention	35(27%)	9(21%)	13(29%)	13(30%)	0.56
Access primary patency rate					
At 3 months	70%	53%	70%	86%	0.004
At 6 months	41%	30%	39%	56%	0.009
At 12 months	27%	21%	29%	30%	0.50
Access secondary patency rate					
At 12 months	93%	94%	86%	98%	0.09
**Clinical event**					
Death (any cause)	6(5%)	1(2%)	3(7%)	2(5%)	0.61
Death (cardiac cause)	3(2%)	1(2%)	2(5%)	0(0%)	0.37
Hospitalization (any cause)	21(16%)	8(19%)	10(23%)	3(7%)	0.12
Hospitalization (cardiac cause)	11(9%)	5(12%)	5(11%)	1(2%)	0.21

Timing of restenosis or re-intervention: early, within 3 months; late, within 4–12 months; P for Chi-square test.

As shown in Figure 3, patients in the lower tertile of EPC received an average of 3.4±2.8 interventions in one year, which was higher than those in the medium tertile (2.6±2.5, P = 0.15) and the upper tertile (1.5±1.5, P = 0.004). As presented in **Table 3**, patients with target-lesion early restenosis had significantly lower CD34^+^KDR^+^ and CD34^+^KDR^+^CD133^+^ cell counts, but not CD34^+^ cell counts, than those with late restenosis.

**Table 3:** Comparisons of EPC levels according to the presence and timing of target-lesion restenosis at one year.

	All patients (N = 130)			Restenosis patients (N = 94)		
EPC	Patent (N = 36)	Restenosis (N = 94)	P value	Late (N = 57)	Early (N = 37)	P value
**EPC (%)**						
CD34^+^	0.062±0.082	0.044±0.047	0.12	0.043±0.038	0.046±0.057	0.77
CD34^+^KDR^+^	0.012±0.017	0.009±0.010	0.33	0.012±0.012	0.005±0.005	<0.001
CD34^+^KDR^+^CD133^+^	0.009±0.017	0.007±0.007	0.27	0.008±0.008	0.004±0.003	<0.001
**EPC (cells/10^5^MNCs)**						
CD34^+^	63±83	45±47	0.13	44±39	46±57	0.89
CD34^+^KDR^+^	9±7	9±10	0.90	12±12	5±5	<0.001
CD34^+^KDR^+^CD133^+^	6±5	6±6	0.85	8±7	4±3	<0.001

EPC, endothelial progenitor cell; MNCs: mononuclear cells.

Timing of restenosis: early restenosis, within 3 months; late, restenosis within 4–12 months.

### Characterization of human EPC and function

**Figure 4:**
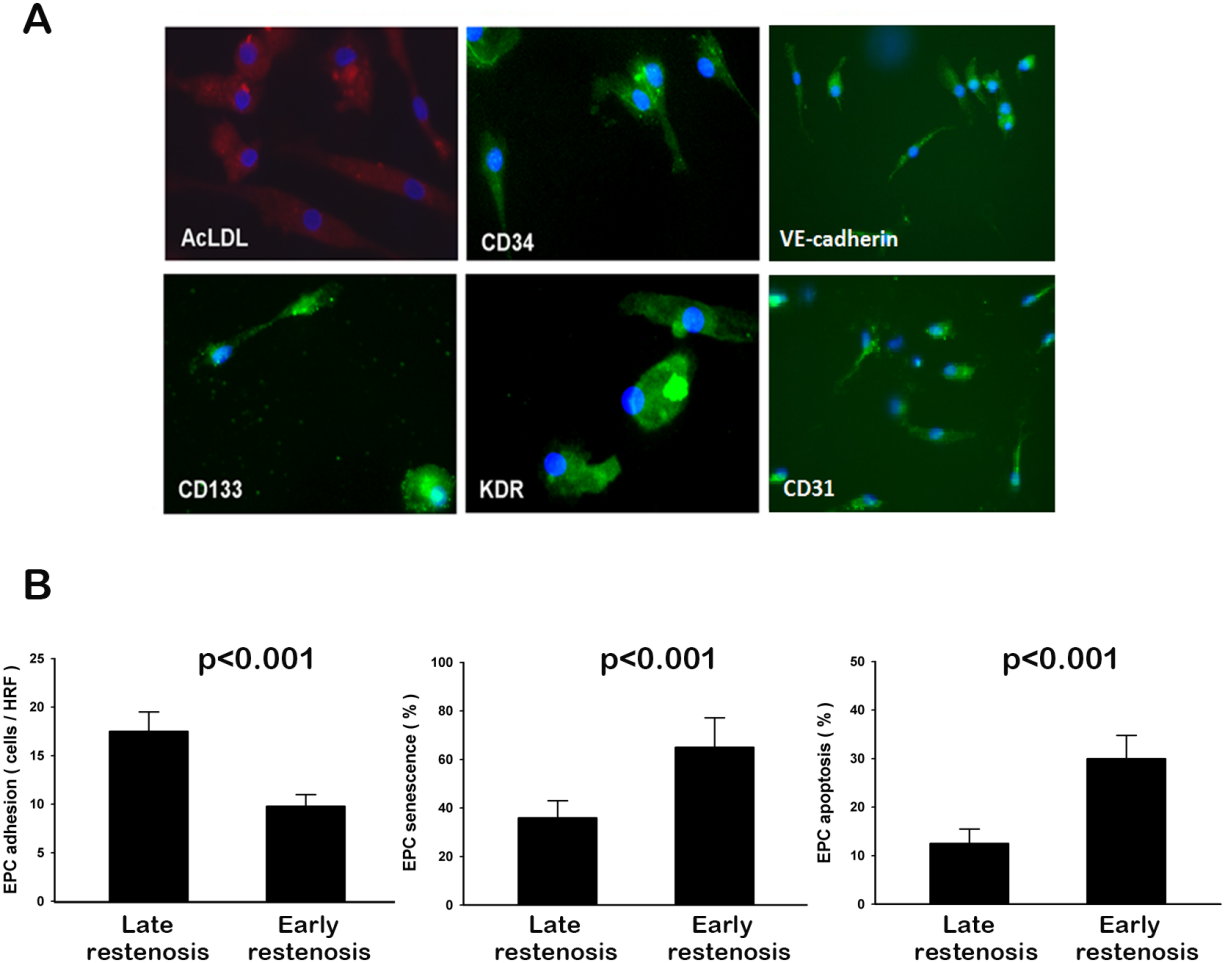
Morphology and functional study. Morphological characterization of human endothelial progenitor cells (EPCs) from peripheral blood (A) and comparisons of the EPC adhesive function (B), senescence assay (C), and apoptosis assay (D) in participants stratified by early (within 3 months) or late (within 4–12 months) restenosis. Values presented as mean ± standard deviation.

The peripheral blood mononuclear cells (MNCs) that were initially seeded on fibronectin-coated wells were round in shape. After the medium was changed on day 4, attached EPCs had an elongated and spindle shape. The EPCs were characterized as adherent cells positive for acetylated low-density lipoprotein (AcLDL) uptake and lectin binding by direct fluorescent staining and immunohistochemical staining. Most of the EPCs expressed endothelial and hematopoietic stem cell markers, CD34, VE-cadherin, CD133, Kinase insert domain receptor (KDR), and CD31 (**Figure 4A**), which are considered critical markers of EPCs.

There were no significant differences in clinical or access characteristics between the two groups of patients, except for lower CD34^+^KDR^+^ and CD34^+^KDR^+^CD133^+^ cell counts in the early restenosis group. (**Table S1**) Patients with early restenosis had attenuated EPC adhesive function compared to those with late restenosis (9.7±2.3 vs. 17.7±3.3 cells/HPF, P<0.001; **Figure 4B**). Patients with early restenosis also had higher percentage of senescence-associated β-galactosidase-positive EPCs (65.5±18.0% vs. 37.6±10.7, P<0.001, **Figure 4C**) and TUNEL-positive EPCs (29.7±8.2% vs. 12.7±5.5, P<0.001; **Figure 4D**) that that with late restenosis.

### Univariate analysis for factors associated with target-lesion early restenosis

In univariate Cox regression analysis, the incidence of target-lesion early restenosis was not associated with demographic factors, cardiovascular risk factors, medications, or biochemical profiles; but significantly associated with access factors, including upper-arm access (hazard ratio [HR], 2.31, 95% confidence interval [CI], 1.09–4.92, p = 0.030), right-sided access (HR, 2.94; CI, 1.03–4.34, p = 0.043), graft access (HR, 2.22; CI, 1.03–4.34, p = 0.020), post-dilatation stenosis (HR, 1.01; 95% CI, 1.00–1.01, p = 0.032), and baseline level of circulating EPCs, including CD34^+^KDR^+^ cell count (HR, 0.91; CI, 0.85–0.98, p = 0.013) or tertiles (high vs. low, HR, 0.20; 95% CI, 0.08–0.53, p = 0.001) and CD34^+^KDR^+^CD133^+^ cell count (HR, 0.89; CI, 0.80–0.98, p = 0.024) or tertiles (high vs. low, HR, 0.25; CI, 0.11–0.59, p = 0.002) (**Table S2**).

### Multivariate analysis for factors associated with target-lesion early restenosis

A multivariate Cox regression analysis with adjustment for covariates confirmed a significant association of CD34^+^KDR^+^ cell tertile (high vs. low, HR, 0.24, CI, 0.08–0.76, p = 0.016) and CD34^+^KDR^+^CD133^+^ cell tertile (high vs. low, HR, 0.23, CI, 0.08–0.64, p = 0.005) with the development of target-lesion early restenosis. In addition, use of calcium channel blockers, side of access and nature of access were identified as independent predictors of early restenosis as well (**Table 4**).

**Table 4:** Multivariate Cox regression analysis for factors predicting target-lesion early restenosis.

Factors	Unit of increase	Hazard Ratio	95% CI	P value
**CD34^+^KDR^+^ cells entered as a continuous variable**				
Use of CCB	Yes vs. no	0.31	0.11–0.87	0.025
Side of access	Left vs. right	0.23	0.07–0.79	0.020
Nature of access	Native vs. graft	0.40	0.18–0.92	0.030
CD34^+^KDR^+^ cells	1 cell/10^5^ MNCs	0.89	0.81–0.97	0.011
**CD34^+^KDR^+^ cells entered as a categorical variable**				
Use of CCB	Yes vs. no	0.33	0.12–0.88	0.027
Side of access	Left vs. right	0.26	0.08–0.88	0.031
Nature of access	Native vs. graft	0.38	0.16–0.86	0.020
CD34^+^KDR^+^ cells	High vs. low	0.24	0.08–0.76	0.016
**CD34^+^KDR^+^CD133^+^cells entered as a continuous variable**				
Use of CCB	Yes vs. no	0.37	0.14–0.99	0.049
Side of access	Left vs. right	0.21	0.06–0.74	0.015
Nature of access	Native vs. graft	0.37	0.16–0.84	0.018
CD34^+^KDR^+^CD133^+^cells	1 cell/10^5^ MNCs	0.82	0.72–0.94	0.003
**CD34^+^KDR^+^CD133^+^cells entered as a categorical variable**				
Use of CCB	Yes vs. no	0.35	0.13–0.94	0.037
Side of access	Left vs. right	0.22	0.06–0.76	0.017
Nature of access	Native vs. graft	0.37	0.16–0.84	0.017
Post-stenosis (%)	1%	1.02	1.00–1.05	0.047
CD34^+^KDR^+^CD133^+^cells	High vs. low	0.23	0.08–0.64	0.005

CCB, calcium channel blocker; CI, confidence interval; MNC, mononuclear cell.

Age, hypertension, diabetes, smoking, dyslipidemia, use of calcium channel blocker, side of access, nature of access, diameter of access, post-angioplasty stenosis were entered as covariates.

## Discussion

### Main findings

Our study showed the impact of circulating EPCs on restenosis of hemodialysis vascular access. Deficiency of CD34^+^KDR^+^ cells was significantly associated with an increased risk of early restenosis. Patients in the lower CD34^+^KDR^+^ cell tertile were four times as likely to experience restenosis compared to those in the upper tertile. In addition, patients with early restenosis were more likely to have impaired EPC adhesive function, and increased cellular apoptosis and senescence. In the clinical setting, uremic patients usually have a cluster of cardiovascular risk factors, which significantly influence the number and function of EPCs. [6] Our study has demonstrated for the first time that there is an association between circulating EPCs and the aggressive venous intimal hyperplasia of hemodialysis vascular access, independent of traditional risk factors.

### Possible mechanisms from animal studies

Angioplasty is associated with mechanical vascular injury, followed by an intensive local inflammatory response, platelet activation, thrombus formation, and intimal hyperplasia. [13] Endothelial disruption is considered to be the primary event in the initiation of restenosis after balloon angioplasty. [14] Besides acting as a mechanical barrier protecting smooth cell migration, a functional endothelium modulates local hemostasis and thrombolysis, and regulates smooth muscle cell proliferation. [14] The importance of endothelial integrity has been demonstrated in animal studies, suggesting that a functionally intact endothelium is requisite for the inhibition of intimal hyperplasia. [14], [15] Accordingly, it is believed that faster re-endothelialization may inhibit the formation of intimal hyperplasia. [16] The traditional paradigm of re-endothelialization is based on the proliferation and migration of pre-existing mature adjacent endothelial cells. Increasing evidence suggests that the injured endothelium is regenerated by circulating EPCs and that the levels of EPCs reflect vascular repair capacity. These cells are derived from the bone marrow and can be mobilized into the peripheral circulation in response to many stimuli, including tissue ischemia and vascular damage, through the release of growth factors and cytokines. [4], [15], [16] In animal studies, Werner et al demonstrated that bone marrow-derived progenitor cells home in to areas of endothelial denudation. [17] Furthermore, intravenous injection of these EPCs can accelerate re-endothelialization and decrease neo-intimal hyperplasia. [18] These animal studies provided mechanical basis for the association between the circulating EPCs and intimal hyperplasia.

### Evidence from clinical studies

In retrospective studies, George et al demonstrated a reduced number and adhesive function of EPCs in patients with proliferative type in-stent restenosis; Matsuo et al also showed reduced EPC numbers and senescence function in patients with in-stent restenosis. [19], [20] In addition, some studies have demonstrated mobilization of EPCs after angioplasty, providing indirect evidence that EPCs may participate in the response to vascular injury. [17] Despite this, conflicting results exist in regards to the relationship between EPC mobilization and subsequent restenosis. [21] Using baseline EPC levels, our study demonstrated that a reduced number of baseline EPCs is an aggravating factor for venous intimal hyperplasia in uremic patients.

Besides being a marker of vascular repair capacity, circulating EPCs are also a surrogate marker for endothelial dysfunction and vascular health. [5] Accordingly, it is possible that deficiency of circulating EPCs may be just a marker of cumulative cardiovascular risk, rather than a direct mediator of intimal hyperplasia. This distinction is important because modulation of EPCs will be beneficial only if it is a ‘disease maker’. Our data favors that EPC deficiency may not only be a marker of intimal hyperplasia for the following reasons. First, most of the cardiovascular risk factors were not correlated with the number of EPCs in our hemodialysis patients. Second, our data is consistent with previous studies demonstrating that cardiovascular risk factors do not predict venous intimal hyperplasia of hemodialysis vascular accesses [22]–[24].

### Early vs. late restenosis

Compared to the 30% restenosis rate after coronary angioplasty [4], the 72% target-lesion restenosis rate in our study confirms the aggressive nature of venous intimal hyperplasia. Although the group with high EPC levels had a better patency rate initially, this difference vanished rapidly within one year. This late catch-up phenomenon suggests that rapid re-endothelialization only delays but does not stop the development of intimal hyperplasia. This is further supported by our observation that no difference in EPC counts between patients with late or no restenosis. Our explanation is biologically plausible as the inflammatory activation and intimal hyperplasia develop earlier than re-endothelialization. According to the animal models of restenosis, the inflammation and cellular proliferation phase occur rapidly after balloon injury, usually within days to weeks. [25] In contrast, the regeneration of the endothelium usually takes weeks to months to complete. [14] In consequence, EPCs seem to play a more significant role in the early stage after angioplasty. After the critical point of re-endothelialization, uremia or hemodialysis-related factors may dominate the development of restenosis, such as endothelial dysfunction, systemic inflammation, repeated punctures of vascular access and less well-defined elastic lamina of veins.

### Comparisons of EPCs of uremic patients between different studies

It is difficult to compare studies because different surface markers or units have been used to measure EPCs. EPC counts in our study were expressed as a fraction of the number of events, rather than events per microliter. We believe this unit is more appropriate for hemodialysis patients because of the substantial variation in fluid status. Although low circulating CD34^+^ cells have been found to be associated with all-cause and cardiovascular mortality in uremic patients [7], we didn’t find a similar association in our study. Furthermore, CD34^+^ cells were not associated with the presence or timing of restenosis in our study as well. This marker is common to a variety of progenitor cells, including smooth muscle progenitor cells, not only endothelial progenitor cells. [26] In a recent animal study using CD34^+^ antibodies to accelerate endothelialization of synthetic grafts, intimal hyperplasia was also stimulated. [27] The dichotomy implies that CD34^+^ antibodies may attract cells with potential transforming into smooth muscle cell or myofibroblasts as well. EPCs represent only a minor cell population in whole blood, and the choice of markers is very important. Because CD34 and KDR are also expressed on circulating mature endothelial cells, surface marker CD133 was used to ensure the identity of EPCs. Despite the rarity of these cells in the circulation, a significant correlation was still observed between counts of these cells and restenosis. There is still confusion about the cell markers used for EPC. The putative EPCs identified by surface marker CD34, KDR, and CD133 in this study are not certainly EPC but also various amounts of hematopoietic stem cells and circulating endothelial cells. These cells could only be discriminated by extensive gene expression analysis or use of a variety of functional assays. [28], [29] Nonetheless, these meticulous methods are not often applied and no simple universal definition exists at the present time. [30], [31] Therefore, putative EPCs in the study were still identified by the panel of CD34, KDR, and CD133 markers, which have been widely used in publications and consistently used as a surrogate marker for cells displaying regenerative properties in human study.

### Limitations

Our study has several limitations that should be considered when interpreting our results. Only pre-procedure EPC levels were measured and the effect of EPC activation, migration, and homing was not evaluated. Second, we were not able to demonstrate increased EPC homing and migration to the injured endothelium, as has been demonstrated in animal studies. Third, there is still no universal definition of EPCs. KDR is not exclusively a marker of EPC but also expressed on hematopoietic stem cells and circulating mature endothelial cells. [29] Various methods to more specifically identify EPCs are emerging but they were not applicable when the study was conducted. [30], [31] Forth, the number of EPCs per 150,000 events analyzed in peripheral blood is relatively low in hemodialysis patients and analysis of more blood amount may be helpful to improve the yield rate. Finally and most importantly, despite our observation of association between EPCs and early restenosis, this is not a sufficient evidence for their causal relationship. Further study to evaluate the effect of EPC manipulation and explore the underlying pathway was needed to prove the mechanical link.

### Clinical implications and conclusion

Although the contribution of EPC deficiency to restenosis remains to be proven, EPC-capturing stents are undergoing clinical evaluation as a coronary intervention. [32] The most relevant clinical implication of our study is that deficiency of certain circulating EPCs is possibly pathogenic for the rapid venous intimal hyperplasia observed in hemodialysis patients. Based on this putative mechanism, methods of modifying EPC number or function, including physical exercise, pharmacological modulation (statin, GSF), infusion of autologous EPCs, capturing EPC to the denudated endothelium, may have the potential to delay the development of restenosis. [13] Studies aimed at modulating the number or function of more specific EPCs is warranted not only to clarify the causal role of EPCs but also as a potential strategy to decrease the frequency. In addition, CD34^+^KDR^+^ cells may serve as a biomarker for patients vulnerable to restenosis. It will be helpful in therapeutic planning, such as aggressive monitoring, EPC-modulating intervention, or early surgical revision. Finally, EPCs seems to play a significant role only in the development of early restenosis. In consequence, therapeutic approach to modulating EPC may focus on this critical period of re-endothelialization.

In conclusion, this study demonstrated for the first time that deficiency of circulating EPCs predicts early restenosis of hemodialysis vascular access. Our observation supports a significant role of circulating EPCs on intimal hyperplasia in human, as that was demonstrated in previous animal models. Further studies to clarify their pathogenic role in human by therapeutic approach are warranted.

## Supporting Information

Table S1 **Characteristics of patients from whom EPC were cultured, stratified by restenosis status.**
(DOC)

Table S2 **Univariate Cox regression analysis for predictors of target-lesion early restenosis.**
(DOC)

Method S1 **Details of Fibronectin adhesion assay, cellular aging assay and apoptosis assay.**
(DOC)
