# Supplementary material for: The Impact of Endothelial Progenitor Cells on Restenosis after Percutaneous Angioplasty of Hemodialysis Vascular Access
Source: PLoS One. 2014 Jun 25;9(6):e101058. doi: 10.1371/journal.pone.0101058 (PMC4071067; doi:10.1371/journal.pone.0101058)
Supplement: Table S1 — Characteristics of patients from whom EPC were cultured, stratified by restenosis status. (DOC) [file pone.0101058.s001.doc]

**Table S1 Characteristics of patients from whom EPC were cultured, stratified by restenosis status.**

| Characteristic | Early  restenosis  (N=10) | Late  restenosis  (N=10) | P  value |
| --- | --- | --- | --- |
| Age (yr) | 64±10 | 64±10 | 0.9 |
| Gender (men/women) | 4/6 | 4/6 | 0.9 |
| Risk factors |  |  |  |
| Hypertension (%) | 8(80%) | 9(90%) | 0.9 |
| Diabetes (%) | 5(50%) | 4(40%) | 0.9 |
| Dyslipidemia (%) | 5(50%) | 4(40%) | 0.9 |
| Current smoker (%) | 2(20%) | 0(0%) | 0.47 |
| Cardiovascular disease (%) | 5(50%) | 3(30%) | 0.65 |
| Biochemical data |  |  |  |
| Cholesterol (mg/dl) | 156±22 | 165±25 | 0.71 |
| Triglycerides (mg/dl) | 177±106 | 147±99 | 0.51 |
| Albumin (g/dl) | 3.8±0.4 | 3.8±0.4 | 0.83 |
| Hemoglobin (g/dl) | 14.0±8.9 | 10.8±2.3 | 0.34 |
| WBC (10^3^/μL) | 7.6±2.7 | 5.8±2.7 | 0.18 |
| Calcium (mg /dl) | 8.9±0.2 | 9.1±0.6 | 0.36 |
| Phosphate (mg/dl) | 4.8±1.2 | 4.5±1.2 | 0.61 |
| Kt/V | 1.40±0.15 | 1.43±0.17 | 0.70 |
| Medications |  |  |  |
| Anti-platelet | 4(40%) | 5(50%) | 0.9 |
| Nitrates | 2(20%) | 2(20%) | 0.9 |
| β-blocker | 2(20%) | 5(50%) | 0.35 |
| Calcium blocker | 1(10%) | 3(30%) | 0.58 |
| ACEI/ARB | 7(70%) | 8(80%) | 0.9 |
| Lipid-lowering agents | 5(50%) | 5(50%) | 0.9 |
| Epoetin (U/kg/week) | 84±29 | 85±25 | 0.9 |
| Access/lesion |  |  |  |
| Shunt age (month) | 70±55 | 57±31 | 0.53 |
| Prosthetic graft | 4(40%) | 4(40%) | 0.9 |
| Upper arm access | 5(50%) | 2(20%) | 0.35 |
| Right arm access | 2(20%) | 2(20%) | 0.9 |
| EPC (cells/10^5^ MNC) |  |  |  |
| CD34^+^ | 50±44 | 62±59 | 0.42 |
| CD34^+^KDR^+^ | 3±3 | 10±8 | 0.02 |
| CD34^+^KDR^+^CD133^+^ | 3±1 | 10±8 | 0.02 |

ACEI, angiotensin converting enzyme inhibitor; ARB, angiotensin receptor blocker; Kt/V, urea clearance; MNC, mononuclear cell; WBC, white blood cell

Timing of restenosis: early, within 3 months; late, within 4-12 months

**Table S2 Univariate Cox regression analysis for predictors of target-lesion early restenosis**

|  | HR (95%CI) | P value |
| --- | --- | --- |
| Age (yr) | 1.01 (0.99-1.04) | 0.43 |
| Male | 1.21 (0.63-2.33) | 0.57 |
| Risk factors |  |  |
| Hypertension | 1.05 (0.55-2.03) | 0.88 |
| Diabetes | 1.25 (0.63-1.33) | 0.53 |
| Smoking | 1.63(0.71-3.74) | 0.25 |
| Dyslipidemia | 0.86(0.38-1.99) | 0.73 |
| Cardiovascular disease | 0.82(0.36-1.89) | 0.64 |
| Laboratory data |  |  |
| Cholesterol >200mg/dl | 0.89 (0.37-2.18) | 0.81 |
| Triglycerides >200mg/dL | 0.89 (0.40-1.98) | 0.77 |
| Albumin | 1.40 (0.27-1.25) | 0.20 |
| Kt/V | 0.45 (0.13-1.55) | 0.21 |
| White blood cell | 0.90 (0.75-1.09) | 0.29 |
| Hemoglobin | 1.06 (0.81-1.38) | 0.67 |
| Medications |  |  |
| Anti-platelet | 0.76 (0.38-1.51) | 0.43 |
| Nitrates | 1.68 (0.79-3.59) | 0.21 |
| β-blocker | 0.93 (0.41-2.13) | 0.86 |
| Calcium blocker | 0.43 (0.18-1.05) | 0.06 |
| ACEI/ARB | 1.70 (0.81-3.55) | 0.21 |
| Lipid-lowering agent | 0.64 (0.23-1.81) | 0.40 |
| Access factors |  |  |
| Shunt age (month) | 1.00 (0.99-1.01) | 0.46 |
| Upper-arm access | 2.31 (1.09-4.92) | 0.03 |
| Right-sided access | 2.94 (1.03-4.34) | 0.04 |
| Graft access | 2.22 (1.03-4.34) | 0.02 |
| Diameter | 1.25 (0.96-1.63) | 0.09 |
| Pre-stenosis (%) | 1.01 (0.99-1.03) | 0.50 |
| Post-stenosis (%) | 1.01 (1.00-1.01) | 0.03 |
| EPC count (cells/10^5^ MNC) |  |  |
| CD34^+^ cells | 1.00(0.99-1.01) | 0.9 |
| CD34^+^KDR^+^ cells | 0.91(0.85-0.98) | 0.01 |
| CD34^+^KDR^+^CD133^+^ cells | 0.89(0.80-0.98) | 0.02 |
| EPC tertile |  |  |
| CD34^+^ cell tertile |  |  |
| Medium vs. low | 0.59 (0.27-1.27) | 0.18 |
| High vs. Low | 0.55 (0.24-1.24) | 0.15 |
| CD34^+^KDR^+^ cell tertile |  |  |
| Medium vs. low | 0.51 (0.25-1.04) | 0.06 |
| High vs. Low | 0.20 (0.08-0.53) | 0.01 |
| CD34^+^KDR^+^CD133^+^ cell tertile |  |  |
| Medium vs. low | 0.41 (0.19-0.87) | 0.02 |
| High vs. Low | 0.25 (0.11-0.59) | 0.01 |

ACEI, angiotensin converting enzyme inhibitor; ARB, angiotensin receptor blocker; Kt/V, urea clearance; MNC, mononuclear cell

**Supplemental Methods:**

**Fibronectin adhesion assay**

EPCs (day 7) were washed with phosphate-buffered saline and gently detached with 0.5 mmol/L EDTA in phosphate-buffered saline. The basic characteristics of these 2 groups were similar. After centrifugation and re-suspension in basal medium with 5% fetal bovine serum, EPCs (1x10^4^ cells) were placed on a fibronectin-coated 6-well plate and incubated for 30 min at 37 ºC. Gentle washing with phosphate-buffered saline was performed 3 times after adhesion for 30 minutes, and adherent cells were counted by independent blinded investigators. Phenotyping of the endothelial characteristics of adherent cells by indirect immunostaining was performed with FITC-labeled lectin from Ulex europaeus (UEA-1). Briefly, the adherent cells were fixed in 2% paraformaldehyde and incubated with 10μg/mL FITC-labeled UEA-1 (Sigma) as previously described.

**Cellular aging assay**

Cellular aging was determined with a Senescence Cell Staining kit (Sigma). Confluent EPCs in 12-well plates were pretreated with microparticles for 4 days. After washing with PBS, EPCs were fixed for 6 minutes in 2% formaldehyde and 0.2% glutaraldehyde in PBS, and then incubated for 12 hours at 37°C without CO2 with fresh X-gal staining solution. After staining, green-stained cells and total cells were counted and the percentage of β-galactosidase-positive cells was calculated.

**Apoptosis assay:**

TUNEL assay (Terminal deoxynucleotidyl transferase mediated deoxyuridine triphosphate nick-end labeling) was performed using the In Situ Cell Death Detection kit (Roche Diagnostics, Basel, Switzerland) according to the instructions of the manufacturers. Confluent EPCs in 12-well plates were pretreated with microparticles for 4 days. Apoptosis was determined as the percentage of positive cells per 1000 DAPI-stained nuclei, and EPCs were visualized under a fluorescence microscope (Nikon Eclipse 50i) at a magnification of 100x.
