# Supplementary material for: The Impact of Endothelial Progenitor Cells on Restenosis after Percutaneous Angioplasty of Hemodialysis Vascular Access
Source: PLoS One. 2014 Jun 25;9(6):e101058. doi: 10.1371/journal.pone.0101058 (PMC4071067; doi:10.1371/journal.pone.0101058)
Supplement: Method S1 — Details of Fibronectin adhesion assay, cellular aging assay and apoptosis assay. (DOC) [file pone.0101058.s003.doc]

**Methods S1**

**Fibronectin adhesion assay**

EPCs (day 7) were washed with phosphate-buffered saline and gently detached with 0.5 mmol/L EDTA in phosphate-buffered saline. The basic characteristics of these 2 groups were similar. After centrifugation and re-suspension in basal medium with 5% fetal bovine serum, EPCs (1x10^4^ cells) were placed on a fibronectin-coated 6-well plate and incubated for 30 min at 37 ºC. Gentle washing with phosphate-buffered saline was performed 3 times after adhesion for 30 minutes, and adherent cells were counted by independent blinded investigators. Phenotyping of the endothelial characteristics of adherent cells by indirect immunostaining was performed with FITC-labeled lectin from Ulex europaeus (UEA-1). Briefly, the adherent cells were fixed in 2% paraformaldehyde and incubated with 10μg/mL FITC-labeled UEA-1 (Sigma) as previously described.

**Cellular aging assay**

Cellular aging was determined with a Senescence Cell Staining kit (Sigma). Confluent EPCs in 12-well plates were pretreated with microparticles for 4 days. After washing with PBS, EPCs were fixed for 6 minutes in 2% formaldehyde and 0.2% glutaraldehyde in PBS, and then incubated for 12 hours at 37°C without CO2 with fresh X-gal staining solution. After staining, green-stained cells and total cells were counted and the percentage of β-galactosidase-positive cells was calculated.

**Apoptosis assay:**

TUNEL assay (Terminal deoxynucleotidyl transferase mediated deoxyuridine triphosphate nick-end labeling) was performed using the In Situ Cell Death Detection kit (Roche Diagnostics, Basel, Switzerland) according to the instructions of the manufacturers. Confluent EPCs in 12-well plates were pretreated with microparticles for 4 days. Apoptosis was determined as the percentage of positive cells per 1000 DAPI-stained nuclei, and EPCs were visualized under a fluorescence microscope (Nikon Eclipse 50i) at a magnification of 100x.
